# Towards scanning nanostructure X-ray microscopy

**DOI:** 10.1107/S1600576723005927

**Published:** 2023-07-28

**Authors:** Anton Kovyakh, Soham Banerjee, Chia-Hao Liu, Christopher J. Wright, Yuguang C. Li, Thomas E. Mallouk, Robert Feidenhans’l, Simon J. L. Billinge

**Affiliations:** a Niels Bohr Institute, University of Copenhagen, Universitetsparken 5, DK-2100 Copenhagen, Denmark; b Department of Applied Physics and Applied Mathematics, Columbia University, New York, NY 10027, USA; c Department of Chemistry, University at Buffalo, The State University of New York, Buffalo, New York, NY 14260, USA; d Department of Chemistry, The University of Pennsylvania, Philadelphia, PA, USA; e European XFEL, D-22869 Schenefeld, Germany; f Condensed Matter Physics and Materials Science Department, Brookhaven National Laboratory, Upton, NY 11973, USA; Australian Synchrotron, ANSTO, Australia

**Keywords:** atomic pair distribution function, nanoparticles, spatial mapping, thin films

## Abstract

This article demonstrates spatial mapping of the local and nanoscale structure of thin film objects using spatially resolved pair distribution function (PDF) analysis of synchrotron X-ray diffraction data. This is exemplified in a lab-on-chip combinatorial array of sample spots containing catalytically interesting nanoparticles deposited from liquid precursors using an ink-jet liquid-handling system. A software implementation is presented of the whole protocol, including an approach for automated data acquisition and analysis using the atomic PDF method. The protocol software can handle semi-automated data reduction, normalization and modeling, with user-defined recipes generating a comprehensive collection of metadata and analysis results. By slicing the collection using included functions, it is possible to build images of different contrast features chosen by the user, giving insights into different aspects of the local structure.

## Abstract 2

A semi-automated workflow is described for rapid-scanning powder X-ray diffraction and pair distribution function experiments. The software infrastructure saves metadata and raw and analyzed files into a collection stored on the local hard drive for easier reuse.

## 1. Introduction

The nanoscale structure of a material has a critical impact on the properties (Billinge & Levin, 2007 ▸). Important nano­structured materials can consist of discrete nanoparticles (Banerjee *et al.*, 2020 ▸), short-range nanostructural modifications to another well ordered structure (Bozin *et al.*, 2019 ▸), nanoporous structures (Jackson *et al.*, 2006 ▸) and so on. In the past few decades, structural tools have emerged for studying nanostructure. When it is static, imaging methods such as transmission electron microscopy (TEM) and scanning tunneling microscopy (STM) can yield direct images of nanostructural features (Jackson *et al.*, 2006 ▸; Turner *et al.*, 2008 ▸). On the other hand, diffraction methods such as the atomic pair distribution function (PDF) analysis of data from powders (Egami & Billinge, 2012 ▸), or from single crystals (Weber & Simonov, 2012 ▸), can yield quantitative nano­structural information (Egami & Billinge, 2012 ▸). Despite this progress, there is still a bottleneck in nanostructure characterization (Richman & Hutchison, 2009 ▸) that requires improved methods, especially in the area of heterogeneous systems.

When samples are heterogeneous, it can be desirable to combine spatially resolved (microscopic/imaging) approaches with diffraction to elucidate spatial variations in local nano­structure. Pioneering work has been done in combining imaging with diffraction in transmission electron microscopes, known as 4D-STEM (Ophus, 2019 ▸; Willhammar *et al.*, 2021 ▸; Johnstone *et al.*, 2020 ▸; Gammer *et al.*, 2018 ▸). Extending 4D-STEM to extract PDFs is also possible (Rakita *et al.*, 2021 ▸; Mu *et al.*, 2016 ▸, 2019 ▸; Liu *et al.*, 2020 ▸).

On the other hand, using X-rays, spatially resolved PDF maps of solid objects have been demonstrated by combining PDF analysis with computed tomography (ctPDF) (Jacques *et al.*, 2013 ▸), for example, mapping nanostructure inside spiral wound AA batteries (Jensen *et al.*, 2015 ▸) and wood cores from the hull of the Mary Rose, a warship of the 16th century king of England, Henry VIII (Jensen *et al.*, 2021 ▸). In general, an important sample geometry is that of a thin film on a substrate. This may be imaged without tomography, similarly to the 4D-STEM described above. Here we explore making spatially resolved nanostructure maps from nanostructured samples on a thin substrate. This is made possible by the recent demonstration that reliable PDFs could be obtained from nano­structured films in normal incidence (tfPDF) (Jensen *et al.*, 2015 ▸), combined with the rapid scanning that is at the heart of the ctPDF development. This could be used, for example, for the analysis of combinatorial arrays of thin film libraries on a chip, a synthesis method that has become a widely accepted industry standard (Service, 1997 ▸; Xiang *et al.*, 1995 ▸) in many fields including heterogeneous catalysis, pharmaceuticals, biomaterials, optics and multi-principal element alloys (Senkan, 1998 ▸; Daly *et al.*, 2015 ▸; Kohn, 2004 ▸; Chan, 2015 ▸; Miracle *et al.*, 2017 ▸; Potyrailo *et al.*, 2011 ▸). We describe here a proof-of-principle experiment along with Python scripts that can be used to handle such spatially resolved data. This shows that high-throughput (HT) scanning probes of nanostructure are possible in thin film geometry, resulting in images of the spatial distribution of different nanostructure parameters such as lattice parameters, atomic positions and atomic displacement parameters, nanocrystallite size and so on. This is distinct from other X-ray microscopy techniques that can give micrometre- or nanometre-scale images, such as ptychography (Pfeiffer, 2018 ▸) and X-ray nanodiffraction (Hrauda *et al.*, 2011 ▸; Cao *et al.*, 2020 ▸), and micro-SAXS (small-angle X-ray scattering) experiments (Liu *et al.*, 2017 ▸), none of which yield PDFs that can be analyzed to extract local structural information versus position, as we describe here. Rather, here we are performing micrometre- and millimetre-resolution spatially resolved measurements of nanostructure extracted from the PDF.

In the lab-on-a-chip experiment one of the key steps is to relate positional information (where the beam hits the sample) with measured data in the form of diffraction images and any prior information from the sample preparation such as target composition. Automation is a priority at modern X-ray synchrotron beamlines where metadata about the instrument configuration, such as motor positions, are available electronically.

Here we describe a protocol, mapPDF, for handling this type of analysis, including data acquisition at the XPD powder diffraction instrument at the National Synchrotron Light Source II, Brookhaven National Laboratory, data reduction that tolerates sample heterogeneity and subsequent data analysis using the PDF technique. The accompanying software allows the data to be reduced and analyzed in a highly automated fashion, and the extracted material-specific properties to be easily visualized as 2D parameter maps.

As a demonstration, we consider an array of catalytic nanoparticles on a carbon paper substrate using an ink-jet-printing approach to allow for deposition of hundreds to thousands of distinct compositions of nanoparticles on a single substrate (Reddington *et al.*, 1998 ▸). We describe the experimental protocol and automated software for carrying out the data analysis and making images that encode the spatial distribution of nanostructural quantities of interest. This supports a major goal in HT nanostructure characterization for situations with hundreds of measurements per hour and analysis times on the same order of magnitude as the measurement time (Potyrailo *et al.*, 2011 ▸). We refer to this approach generically as scanning nanostructure X-ray microscopy (SNXM).

The protocol is developed for screening spatially resolved PDF data and is modular in design. This allows the protocol to be extended to a wide variety of HT experiments, such as *in situ* synthesis experiments, as well as other experimental techniques.

## 2. Experimental

### 2.1. Sample preparation

**Figure 1:**
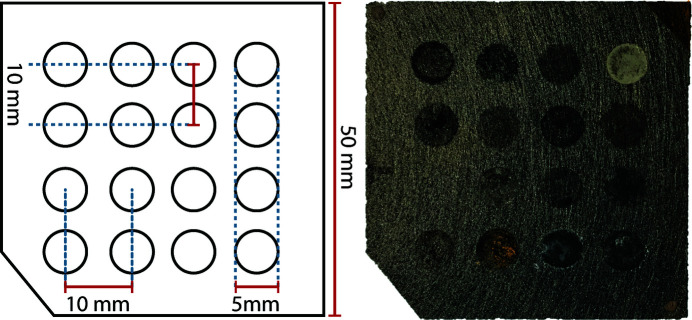
A typical sample layout for combinatorial studies (left) and the tested array for catalytic material (right). A square piece of carbon paper was used as a substrate for the ink-jet-printed material in a 4 × 4 configuration.

The combinatorial catalyst library was deposited using a Pipetmax automated liquid-handling system on semicrystalline carbon paper (Toray 120, from FuelCellStore) in a 4 × 4 grid, giving 16 circular deposition sites (‘wells’) of 5 mm in diameter and with a center-to-center spacing of 10 mm (Hitt *et al.*, 2021 ▸). Transition metal nitrate solutions at 0.1*M* were used for deposition, except for the Au well where HAuCl_4_ was used. The precursor solutions were mixed onto the carbon paper and reduced with excess hydrazine solution. The sample was then vacuum-dried overnight in a 60°C oven and washed with deionized water to create the differently alloyed metal samples on the substrate, as shown in Fig. 1 ▸. The choice of chemicals, size, number of samples and pattern is programmable from the liquid-handling system for future implementations of this protocol.

### 2.2. Synchrotron X-ray measurements

**Figure 2:**
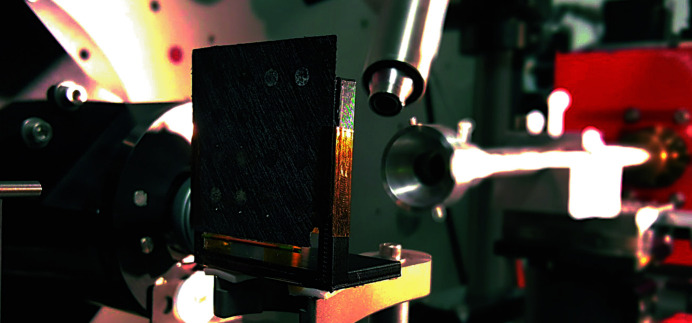
The combinatorial library mounted on a 3D-printed bracket in front of the X-ray beam. The array is mounted on the goniometer which allows measurement access to all deposition sites.

The experiments were carried out at the 28-ID-2 (XPD) beamline at NSLS-II, using the normal incidence thin film PDF method (Jensen *et al.*, 2015 ▸). The combinatorial array was mounted perpendicular to the X-ray beam direction using a 3D-printed bracket. The measurements were performed in a transmission geometry, as shown in Fig. 2 ▸.

The array was moved using goniometer motors in an *xy* plane perpendicular to the incident beam direction, with a fixed sample-to-detector distance. The 2D PerkinElmer detector was placed behind the sample at a distance of 203.4 mm, which gave an effective instrumental *Q* range, where 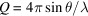 , of 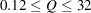 Å^−1^. The incident wavelength of the X-rays was λ = 0.183983 Å with a beam cross section at the sample of 250 × 300 µm in the vertical and horizontal directions, respectively.

The sample wells are much larger than the beam, and the sample distribution within the wells is not uniform (see Section 3). We therefore sought a measurement protocol that scanned over large areas of the sample in order to find the best measurement conditions for sample determination, and also assess the heterogeneity of the sample. A zoomed-in measurement area of 9 × 15 mm was chosen, over which the beam was scanned in a snake scan pattern where the sample is scanned horizontally and then the direction of the scan is reversed after a small vertical offset. The chosen scan pattern encompassed two catalyst ‘wells’ containing AuAg and AgCu nanocrystalline material, as shown in Fig. 1 ▸.

A coarse alignment was done to set the position of the first measurement point by using a laser coaxially aligned with the incoming X-ray beam. The snake measurement pattern was then performed with a series of 1 mm steps executed vertically, followed by a 1 mm horizontal offset, followed by 1 mm vertical steps in the opposite direction, repeated to cover the full measurement area. Note that, since the beam size was 250 × 300 µm, the full area is being undersampled. In the current case, the positional resolution of the stepper motor was much finer than the beam size or step size, but if these values become small it is important to make sure that experiments are carried out in a regime where the stepping accuracy of the positional motors is much better than these other parameters. The exposure time was selected on the basis of signal quality from a preliminary measurement on a nanoparticle spot, and set to 5 s per point, resulting in a measurement throughput of over 6000 measurements per hour.

The sample–detector distance, *Q* range and geometric orientation of the detector were calibrated by measuring a crystalline Ni powder mounted on the same bracket that holds the sample chip prior to data collection from the sample itself. The experimental geometry parameters were refined using the *FIT2D* program (Hammersley, 2004 ▸). A mask was created to remove outlier pixels (dead pixels, hot pixels and pixels shadowed by the beamstop) and applied to the 2D images from the measurement series, before carrying out the azimuthal integration to a 1D diffraction pattern.

The carbon sheet produces a significant background signal in this experimental geometry, but the background signal can be subtracted from the data, leaving only the structural information of the deposited material. We found that background subtraction is not trivial for these samples and we developed a protocol for doing it that is described in the next section. The total scattering structure function, *F*(*Q*), was then obtained after standard corrections and normalizations of the data, and Fourier transformed to obtain the PDF, using *PDFgetX3* (Juhás *et al.*, 2013 ▸) within *xPDFsuite* (Yang *et al.*, 2014 ▸). The maximum range of data used in the Fourier transform ( 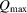 ) was chosen to be 21 Å^−1^ in the current case, which was the best compromise between real-space resolution of the PDF signal and noise.

## 3. Results

### 3.1. Protocol automation software

The main goal of the protocol is to address the large number of measured data points that are generated during HT experiments. We have written a set of Python scripts that are intended to be highly flexible and customizable, allowing for efficient data collection, curation, reduction and analysis. The software is intended to be accessible and user friendly. The code can be executed using *IPython* (Perez & Granger, 2007 ▸) and *Jupyter* notebooks.

**Figure 3:**
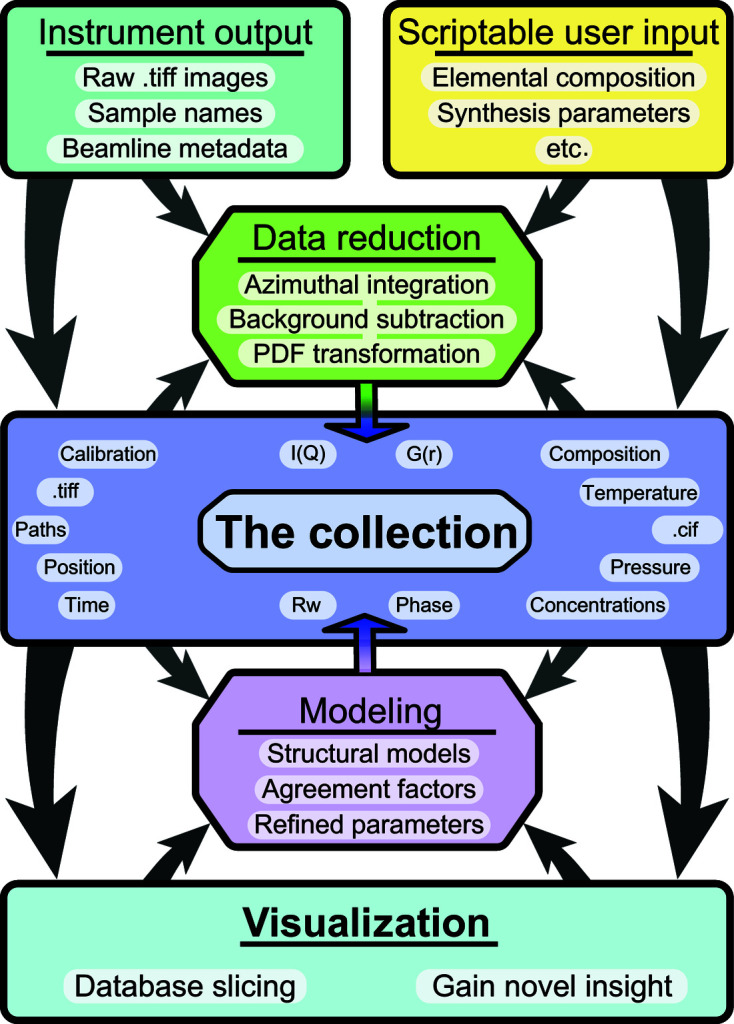
A flowchart illustrating the core of the mapPDF protocol and current implementation. Instrumental output is combined with user-created metadata to perform data reduction. Every step of the process is saved in the collection, which can be sliced and visualized for screening and advanced analysis.

The overall approach builds a collection of information about the experiment, associating reduced data, user inputs based on prior knowledge of the material and analysis results. The collection can then be interrogated and visualized easily by the user to draw conclusions from the parts of the entire data set. For example, using the language of *HyperSpy* (Pena *et al.*, 2017 ▸), the data set may consist of a 1D spectrum [for example, the *F*(*Q*) function or the PDF, *G*(*r*)] at each location in a 2D grid of positions, and these can be thought of as a 1D ‘signal dimension’ and two ‘navigation dimensions’. It is then possible to plot some quantity derived from the signals in a false-color plot over the navigation dimensions. A schematic of the overall layout is given in Fig. 3 ▸, showing all of the modules and the general workflow.

The data analysis protocol is currently optimized for the XPD beamline at NSLS-II. After a measurement, the acquisition software at the beamline outputs a log file containing the metadata, such as motor positions, measurement times and unique identifiers for each diffraction image.

In the first step, the protocol software interrogates the log file and converts each measurement entry into an event. Each event then contains links to positional and other measurement metadata and the corresponding image files. The main benefit of the approach is manageability of the contents of the collection, which are easy to visualize for the user using standard Python plotting packages such as *matplotlib* (Hunter, 2007 ▸) in conventional 1D or heatmap plots by simple iteration and filtering of the corresponding keywords. In addition, we have prepared a few custom plotting functions that produce the figures presented.

Any pre-existing knowledge can be appended to the corresponding event entries using simple macros, using *e.g.* Python for loops and conditional statements. For example, we can add composition information based on our prior knowledge of the layout of the sample:[Chem scheme1] 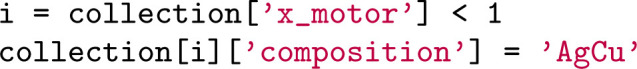


This way, any useful information which is absent in the metadata can be added on a per entry basis.

Experimental geometry calibration information is obtained from a Ni standard material measured at the same time as the array (Fig. 2 ▸). Calibration parameters are used when the images are azimuthally integrated to 1D *I*(*Q*) patterns using *FIT2D* (Hammersley, 2016 ▸) or *PyFai* (Ashiotis *et al.*, 2015 ▸). The integrated patterns are then linked alongside the other information in the collection to the correct events as data arrays.

The different steps of the data acquisition, processing and modeling protocols are described in more detail below. Here we take a high-level view of how the information is shared between different parts of the program, referring to the flowchart in Fig. 3 ▸. The user creates text files, ‘Scriptable user input’, with metadata about the experiment. Additional metadata about the experiment may come from the measuring instrument. For example, at the 28-ID-2 XPD beamline at NSLS-II the data acquisition software uses the *xpdAcq* (https://github.com/xpdAcq/xpdAcq) package built on Bluesky (Arkilic *et al.*, 2017 ▸), which saves diffraction images on the file system in tiff format with user-supplied filenames. It also saves other metadata about the experimental setup in a database using the databroker (Arkilic *et al.*, 2017 ▸) infrastructure. This is shown in the ‘Instrument output’ panel in the diagram, which shows schematically how user-supplied input and ‘experiment’-supplied information, whatever its origin, may be fed into the system. ‘Data reduction’ shows the steps that are taken to reduce the data from raw images all the way to 1D powder diffraction patterns and PDFs. In general, these steps require information from the user and the experiment, as indicated by the arrows. ‘The collection’ is the central core of the system and represents a living database of information that is captured and stored about the entire experiment. To begin with, it just contains the raw experimental and user-supplied information, as indicated by the outer downward arrows. Desired metadata that were stored in user- and experiment-supplied text files and databases are stored in the collection for easier reuse. The upward arrows to the data reduction panel indicate that data reduction parameters may be pulled either from the collection database or from user-supplied scripts. After a data reduction has been performed, the results of the reduction are then appended to the collection, as indicated by the vertical downward arrow. At this point, the user may want to carry out a visualization of the reduced (but not yet modeled) data. This is possible via the visualization engine which uses metadata in the collection to map parameters of interest to the user from the reduced data [we refer to these as ‘quantities of interest (QoI)’] into spatially resolved maps; the user is also able to select individual pixels for closer examination to plot data at different stages of the processing. Finally, since we are chaining analysis steps, we can actually carry out modeling on the reduced data using the *DiffPy-CMI* modeling engine, which is shown in the ‘Modeling’ panel. The modeling programs take reduced data and model information from the collection and carry out regression, resulting in best-fit models. Structural parameters obtained from these models may then be mapped as QoIs and the fits explored in greater detail for individual pixels. The results of the modeling are then also deposited in the collection, which becomes a self-contained history of the experiment and data analysis and modeling campaign.

### 3.2. Background subtraction

The tfPDF measurement requires careful subtraction of the substrate scattering because the substrate signal (background) was significant compared with the small signal from the deposited nanoparticles.

Background images were acquired from a sample region with no nanomaterial and integrated to *I*(*Q*) in the same way as the images containing the material. A different background measurement can be assigned to each entry in the collection. This is particularly useful when substrate properties vary as a function of position and a background measurement in proximity to the material of interest is optimal for signal extraction. In the present case, a single background data set was collected from the center of the array and assigned to all images.

The background subtraction is performed after interpolating the background data set onto the *Q* grid of the target pattern. In most cases, a scaling factor of 1 is used for all backgrounds in a data set, but a global scale factor may be defined by the user, if needed. Additionally, a utility function has been provided to optimize the background subtraction for each entry in the collection, by minimizing the difference between sample and background signal intensity over a user-defined *Q* range (Jacques *et al.*, 2013 ▸).

The background-subtracted diffraction patterns are then appended to the collection as data arrays.

### 3.3. PDF transformation and model fitting

**Figure 4:**
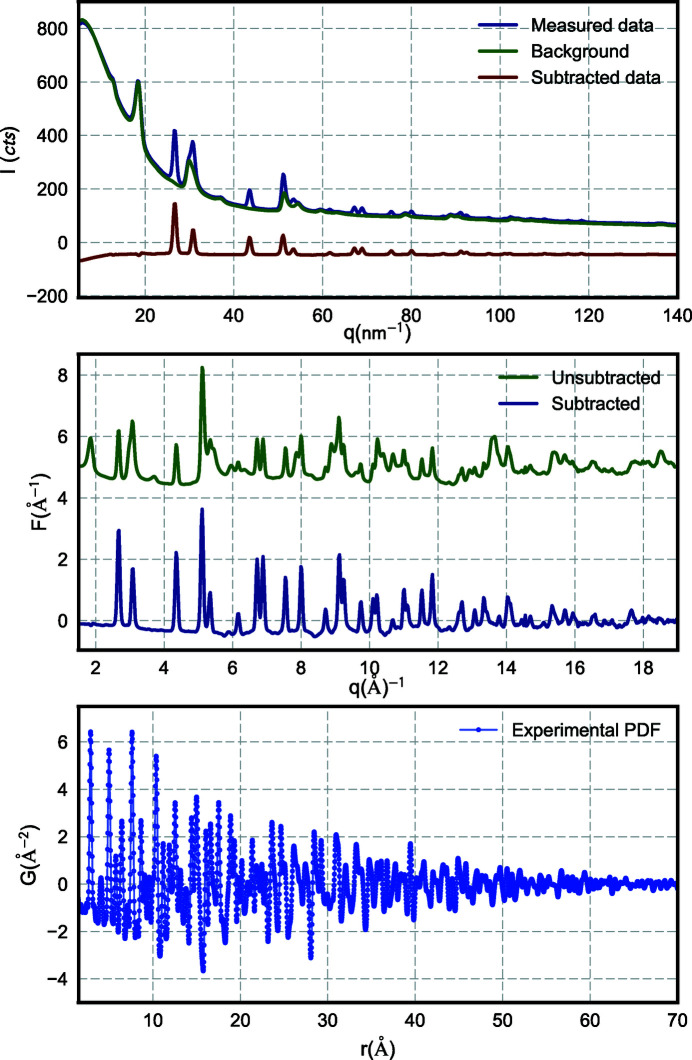
An example of data processing from a single event in the collection. The background signal is subtracted following normalization in order to better resolve scattered intensity from the nanoparticle sample.

The background-subtracted *I*(*Q*) data are Fourier transformed to the PDF with *PDFgetX3* (Juhás *et al.*, 2013 ▸) using parameters such as 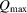 and elemental compositions that are chosen by the user and then stored in the collection. The output PDF data, *G*(*r*), are again appended to the main collection. A representative example of data at each step of the process is shown in Fig. 4 ▸. These transformation steps can be performed on all database entries or a subset.

**Figure 5:**
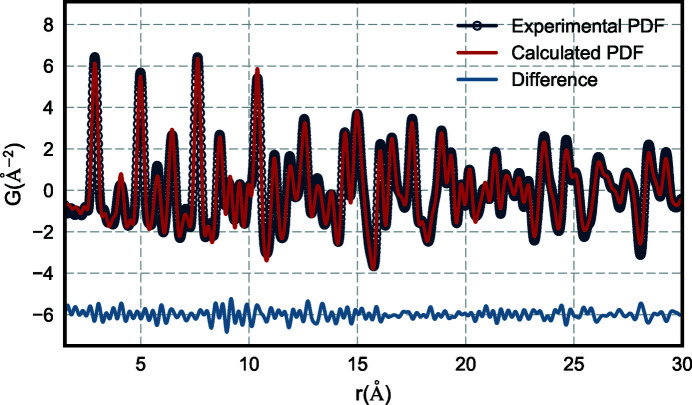
An example of a single PDF fit using a bimetallic f.c.c. model to one of the data files in the collection. The blue symbols illustrate the experimental data. The refinement score ( 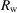 ) is 12.3%.

In the combinatorial array experiment presented, each well contained different metallic nanoparticles. We used a face-centered cubic (f.c.c.) model to refine the experimental PDFs and extract structural parameters for each event in the collection. The PDFs, relevant metadata entries and initial guesses for the structural parameters are fed into the model to perform structural refinement using *DiffPy-CMI* (Juhás *et al.*, 2015 ▸), complex modeling infrastructure software available at https://www.diffpy.org/. A representative example from the combinatorial array experiment may be seen in Fig. 5 ▸. In this example, the model used was based on the close-packed f.c.c. nickel structure. Although the nanoparticles were alloys, we assumed a random, chemically disordered f.c.c. structure where the different average compositions could be accounted for by the average scale factor for the phase and variations in the lattice parameters. This is a simple but effective approximation that is robust for fitting across the different compositions. The refined variables were the phase scale factor, lattice parameters, a single isotropic atomic displacement parameter and *spdiameter*, a spherical particle diameter parameter. The diffraction profile parameters 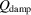 and 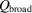 were found by fitting a reference PDF from bulk nickel measured under the same conditions, and fixed during the nanoparticle refinements. The primary parameters of interest from the output of the refinement for this data set, namely the crystallite size and lattice parameter, and weighted agreement factor 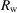 , are associated with the correct event in the collection, as shown in Fig. 3 ▸.

Although not illustrated here, other powerful analyses based on AI and machine learning (ML), which sort and cluster data, may be carried out, such as non-negative matrix factorization (NMF) (Liu *et al.*, 2021 ▸; Geddes *et al.*, 2019 ▸) or principal component analysis (Chapman *et al.*, 2015 ▸); or one may use similarity measures such as the Pearson correlation coefficient metric (Yang *et al.*, 2014 ▸; Jensen *et al.*, 2021 ▸), which can automatically cluster or find component signals patterns that are the basis for large sets of data such as position- or time-sensitive data. The flexibility of the framework is ideal for incorporating such analyses using easy-to-use functions from widely used Python libraries such as *scikit learn* (Pedregosa *et al.*, 2011 ▸).

### 3.4. Visualizing spatially resolved data

Good visualization tools are essential for HT experiments. The approach outlined above results in a comprehensive collection of measured data and data analysis results. Presenting these data in a manageable way is usually a major challenge.

**Figure 6:**
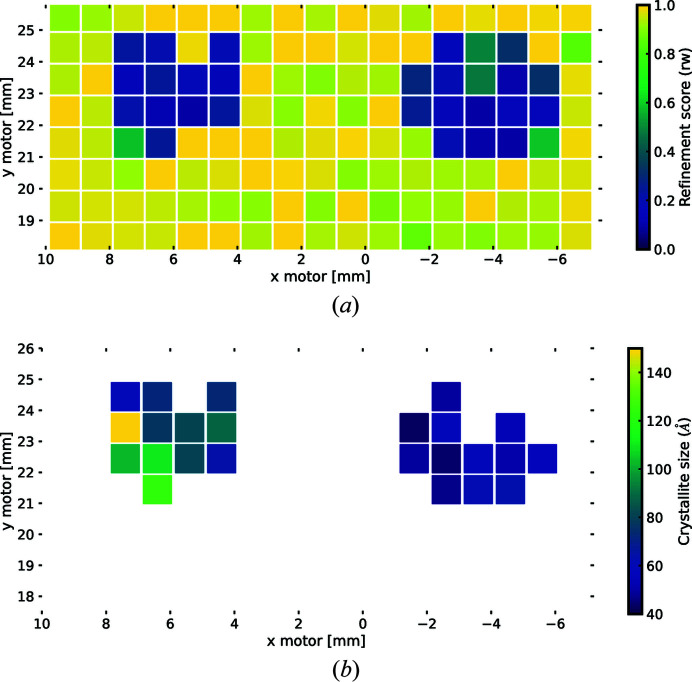
(*a*) Map of refinement scores versus position for the array. Yellow squares indicate a measurement area with a poor refinement score, while blue squares indicate areas with good refinement scores, and thus presence of the f.c.c. phase. There are two distinct regions with nanomaterial surrounded by measurements of nothing but the background. The refinement scores for this data set are highly correlated with the signal-to-noise ratio and give an indirect metric for the amount of material in a given area. (*b*) Map of particle size versus position on the array filtered to only display good model refinement scores. The color scale indicates the spherical particle diameter parameter from smaller (blue) to larger (yellow) crystallite size estimates. The figures are generated using simple conditional statements to slice the collection.

Our main philosophy is to make spatial maps of scalar quantities that are associated with some aspect of the components in the collection, for example, goodness of fit or lattice parameter. In other words, we extract a scalar from the signal dimension and plot it against the navigation dimensions. Fig. 6 ▸ illustrates a use-case where the position of the quantity on the plot corresponds to the physical position on the chip where the data were measured, as viewed along the direction of travel of the X-rays.

Fig. 6 ▸(*a*) shows the 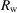 from fits of the f.c.c. model to the background-subtracted data from our array of catalytic material as a function of position on the array. The plot can be generated from a complete collection using a simple plotting function:[Chem scheme2] 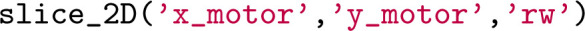


This function loops over the collection, extracts the parameter of interest, sets the correct boundaries and sets a color scale for the false-color plot. The color of the squares indicates the model fit quality at the measured position, where yellow indicates minimal or no agreement with the candidate structure and dark blue indicates good agreement. After the background-subtraction step, the areas where all of the signal is from the substrate would contain nothing but noise. Since we are fitting the f.c.c. model, these regions will result in poor 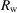 values and good fits are an indication of where the catalytic material is located and how much is there.

We can then return to the locations exhibiting a better fit, which contain signal from the material of interest, to do more careful structural analyses. Note that, in the current case, the beam size is smaller than the step size and so our 2D navigation space is being undersampled. In general, this is a choice that is made at data collection time and is a trade-off between spatial resolution of the measurement, measurement area, time available and the characteristics of the beam. We have not explored this phase space in detail, but the software tools allow preliminary measurements to be quickly collected and visualized to aid in making this decision early in the experiment.

In a similar fashion to Fig. 6(*a*), it is possible to generate maps [like the one presented in Fig. 6 ▸(*b*)] of any quantity in the collection with multiple filters by using simple Python for loops, conditional statements and built-in *matplotlib* functions:[Chem scheme3] 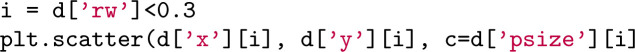


The code snippet above generates the spatial map of nanoparticle size versus position refined from the f.c.c. model after filtering for an acceptable 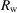 threshold. From the figure, it becomes clear that the particle size distributions differ within the wells, with the AgAu well being much more uniform and smaller on average.

All outputs may be saved and stored for later use. Currently, by default, data are saved in comma-separated value (c.s.v.) files which are very easily reloaded into the *Pandas dataframe* (McKinney, 2011 ▸) objects that hold the data during the analysis. However, the exact form of the output on the file system can be modified in the future, for example using structured binary formats such as hdf5 and even storing results in databases such as *mongoDB* (Banker *et al.*, 2016 ▸).

### 3.5. Software flexibility, modularity and availability

The software that implements the protocol can be divided into several key parts as presented in Fig. 3 ▸. These are initial data treatment, transformation of the data, and model fitting and refinement. Because of the modularity, all three can be modified, replaced or omitted by the user, depending on the use-case and user preferences, allowing the user to easily build a bespoke analysis for their data.

Although originally intended for tracking positional information about the sample, the protocol can be extended to keep track of any scalar quantity and has been found to be extremely useful for time-series data sets. Structural parameter evolution as a function of time, instead of being a function of motor positions, can be visualized using the protocol software which helps streamline systematic analyses of large *in situ* data sets, for example. The methodology is currently being extended for studying nanocluster formation in a wet synthesis environment measured at the P.02 beamline at PETRA III, Hamburg, Germany.

The latest source code is available as open source with a BSD license on GitHub as part of the DiffPy organization at https://github.com/diffpy/diffpy.mappdf; also available is an example data set used to generate the figures above. We welcome contributions that will extend the code. For example, in principle, it would be possible to add a Rietveld refinement capability to augment the *DiffPy-CMI* modeling of the PDF data, extending it to accept scanning electron PDF data (Rakita *et al.*, 2021 ▸), or other similar enhancements.

## 4. Conclusion

An analysis protocol and a set of scripts for treating a wide variety of combinatorial high-throughput materials characterization data are presented. The protocol software is flexible and can be modified and expanded by the user. An example of a combinatorial catalyst library analyzed using the PDF technique has been demonstrated, highlighting the power of the approach. The ability to rapidly analyze and visualize large volumes of spatially (or time or some other independent variables) resolved data greatly facilitates high-throughput experiments at synchrotron sources.
